# Myeloid Cell Sirtuin-1 Expression Does Not Alter Host Immune Responses to Gram-Negative Endotoxemia or Gram-Positive Bacterial Infection

**DOI:** 10.1371/journal.pone.0084481

**Published:** 2013-12-26

**Authors:** Laura E. Crotty Alexander, Brenda J. Marsh, Anjuli M. Timmer, Ann E. Lin, Kayvan Zainabadi, Agnieszka Czopik, Leonard Guarente, Victor Nizet

**Affiliations:** 1 Pulmonary Critical Care Section, Veterans Affairs San Diego Healthcare System, San Diego, California, United States of America; 2 Department of Medicine, University of California San Diego, San Diego, California, United States of America; 3 Department of Pediatrics, University of California San Diego, San Diego, California, United States of America; 4 Department of Biology, Massachusetts Institute of Technology, Cambridge, Massachusetts, United States of America; 5 Skaggs School of Pharmacy and Pharmaceutical Sciences, University of California San Diego, San Diego, California, United States of America; National Institute of Environmental Health Sciences, United States of America

## Abstract

The role of sirtuin-1 (SIRT1) in innate immunity, and in particular the influence of SIRT1 on antimicrobial defense against infection, has yet to be reported but is important to define since SIRT1 inhibitors are being investigated as therapeutic agents in the treatment of cancer, Huntington’s disease, and autoimmune diseases. Given the therapeutic potential of SIRT1 suppression, we sought to characterize the role of SIRT1 in host defense. Utilizing both pharmacologic methods and a genetic knockout, we demonstrate that SIRT1 expression has little influence on macrophage and neutrophil antimicrobial functions. Myeloid SIRT1 expression does not change mortality in gram-negative toxin-induced shock or gram-positive bacteremia, suggesting that therapeutic suppression of SIRT1 may be done safely without suppression of myeloid cell-specific immune responses to severe bacterial infections.

## Introduction

Sirtuin 1 (SIRT1) is an evolutionarily conserved member of the sirtuin family of NAD^+^-dependent deacetylases. Since their discovery in 1999, the sirtuins have been the subject of intensive scientific investigation and discussion. Notably, SIRT1 was implicated as a mediator of the life-extending effects of calorie restriction [1–3] and over-expression of SIRT1 was thought to have life-extending effects of its own [4–6]. Both of these findings have been challenged in subsequent analyses [7–9] and the true role of SIRT1 in lifespan regulation remains controversial. Regardless of their life-extending properties, sirtuins have been shown to play a complex and critical role in metabolism and cellular stress responses, and as such are being investigated as therapeutic targets in diabetes, cardiovascular diseases, inflammatory conditions and neurodegenerative disorders [10].

Recent evidence has shown that SIRT1 plays a multifaceted role in adaptive immunity via suppression or amplification of T and B cell inflammatory responses in a context-dependent manner. Upregulation of SIRT1 *in vitro* is associated with T cell anergy and decreased response to IL-2 [11], but suppression of SIRT1 promotes the expression of Foxp3 in regulatory T cells (Tregs), thereby amplifying their immunosuppressive activity *in vitro* and *in vivo* [12,13]. SIRT1 knockout (KO) mice develop eyelid inflammation in infancy [14], and a lupus-like nephritis at later ages [15]. However, suppression of SIRT1 in an established mouse model of lupus (MRL/lrp mice) decreases autoantibody production and renal pathology [16]. Thus, SIRT1 has complex roles in immune responses and autoimmunity, and both pharmacologic and genetic tools are important in delineating the specific inflammatory mechanisms regulated by SIRT1. 

To date, little is known about the function of SIRT1 in innate immunity and host defense. Whereas several studies have indicated that SIRT1 suppresses innate inflammatory responses [17–19] others have reached the opposite conclusion [20,21]. The influence of SIRT1 on antimicrobial defense against infection has yet to be reported but is important to define, particularly since SIRT1 inhibitors are being investigated as therapeutic agents for the treatment of Huntington’s disease and cancer ([22–25]; clinical trials NCT01521832 and NCT01521585). Other molecular inhibitors used to treat these disorders have led to immunodeficient states and increased susceptibility to infection. 

Given the previous controversies regarding the role of sirtuins in mammalian systems, we examined in detail the consequence of genetic depletion and pharmacological SIRT1 modulation on leukocyte and whole animal responses to invasive bacterial infection and lipopolysaccharide (LPS)-induced endotoxemia.

## Materials and Methods

### Ethics Statement

This study strictly adhered to the recommendations in the Guide for the Care and Use of Laboratory Animals of the National Institutes of Health. Ethics approval for animal experimentation was obtained from the Institutional Animal Care and Use Committee of the University of California at San Diego, USA (Protocol Number: s00227m). All efforts were made to minimize suffering.

### Cell Lines, Bacterial Strains, and Reagents

Human-derived HL60 promyelocytic cells and mouse-derived RAW 264.7 macrophage (Mφ) cell lines (ATCC) were utilized for *in vitro* pharmacologic experiments. Cell lines were grown in RPMI-1640 with 10% FBS at 37°C and 5% CO_2_. DMSO (1.25%) was added to the media of HL60 cells 5 days prior to use in assays to induce neutrophilic differentiation. Experiments were run in RPMI-1640 with 2% FBS at 37°C and 5% CO_2_. Group B Streptococcus (GBS) COH1 strain was grown to mid-log phase (absorbance at 600 nm = 0.4) in Todd-Hewitt broth (THB) at 37°C, resuspended in PBS to 1 x 10^8^ cfu/ml, and mammalian cells were infected at a multiplicity of infection (MOI) of 0.5 and 0.1. THB with yeast extract (THY) was inoculated with Streptococcus pneumoniae (SPN) strain D39 at a 1:10 dilution, and grown to mid-log phase at 37°C with 5% CO_2_, resuspended in phosphate-buffered saline (PBS) to 2 x 10^8^ CFU/ml, and mammalian cells were infected at an MOI of 0.1. The putative Sirt-1 activator resveratrol and Sirt-1 inhibitor sirtinol (Sigma) were used for pharmacologic studies. LPS from *Escherichia coli* O111:B4 (Alexis) was used for *in vivo* endotoxemia studies. ELISA Duosets from R&D Systems were utilized for cytokine analysis of serum from mice.

### Mouse Strains and Primary Cell Isolation

Myeloid-specific SIRT1 KO (lysMcre Cre+ SIRT1^flox/flox^) mice in a C57BL/6 background and WT (lysMcre Cre- SIRT1^flox/flox^) mice were used for all experiments. Mice were age (6-9 months) and sex-matched for all experiments. Mice were sedated with inhaled isoflurane and terminally bled. Blood from 5 mice was pooled for assays. Peritoneal Mφs were isolated by peritoneal lavage with 10 mL PBS, repeated five times per mouse, and Mφs were pooled from 3-5 mice. Peritoneal neutrophils were harvested 4 hours after intraperitoneal (IP) injection of 500 μL of thioglycolate, in a similar fashion. Alveolar Mφs were harvested by lavaging the airways five times with 800 μl PBS. Bone marrow (BM) was harvested as previously described [26]. In brief, femurs and tibias were flushed and BM was resuspended in water for 10 seconds to lyse red blood cells. To derive Mφs, BM cells were grown in RPMI-1640 with 20% FBS, 25% L-cell supernatant, 100 IU/ml penicillin, and 100 μg/mL streptomycin at 37°C and 5% CO_2_. BM Mφs were harvested with EDTA on day 6 and re-plated at 1 x 10^5^ cells/well in 96 well plates, and assays were run on day 8. For BM neutrophils, BM was resuspended with 5 ml PBS and layered on top of 5 ml each Histopaque 1119 and 1077, spun at 1200 rpm for 30 minutes at RT and the layer of cells at the intersection of the two Ficoll layers was resuspended in RPMI-1640 with 5% FBS and 1 μg/ml PMA, plated at 1 x 10^5^ cells per well in 96 well plates, and incubated at 37°C with 5% CO_2_ for 10 hours prior to running experiments. Samples of all myeloid cells (1 x 10^6^ in triplicate) were placed in TRIZOL (Invitrogen) and frozen at -80°C. Samples were rapidly thawed and total RNA was isolated using the standard TRIZOL RNA isolation protocol (Invitrogen). RNA was treated with TURBO^TM^ RNase-free DNase (Ambion®, Life Technologies) and cDNA was synthesized from total RNA by iScript cDNA synthesis kit (BioRad). cDNA underwent quantitative PCR (qPCR) analysis with SIRT1 primers (Jackson Labs: F and R) using the KAPA SYBR® FAST qPCR kit (KAPA Biosystems). Efficiencies of all primers were within 85-100%. mRNA abundance was obtained by normalizing to beta-tubulin levels.

### Bactericidal Assays

HL60 cells, RAW cells, Mφs and neutrophils were plated at 3 x 10^5^ cells/well in 24-well plates (Falcon) the day prior to infection. For pharmacologic studies, cells were incubated with increasing concentrations (5, 10, or 20 μM) of resveratrol, a SIRT1 activator, or sirtinol (10 μM), a SIRT1 inhibitor, for 24 hours prior to infection. At given time points the number of surviving bacteria in each well (extracellular and intracellular) was determined by adding triton to a final concentration of 0.025% to each well to lyse mammalian cells, followed by serial dilution and plating for enumeration of bacteria. Whole blood assays were done as previously described [27]. In brief, 300 μL of whole blood was placed in 2 mL siliconized tubes (Fisher) and 100 μL of log-phase bacteria was added to a final concentration of 1 x 10^4^ CFU/mL. Tubes were incubated at 37°C on a rotator, and at various time points 25 μL was taken and serially diluted in sterile water to lyse mammalian cells, and plated for enumeration of surviving bacteria per mL. Phagocytosis of SPN was determined by incubating WT and KO BM Mφs with SPN for one hour at 37°C, rinsing with warm PBS three times, applying antibiotics (penicillin 10 ug/mL and gentamycin 100ug/mL) for 60 minutes, followed by rinsing with warm PBS three times, lysis with 0.025% triton, serial dilution, plating, and enumeration of CFU. Intracellular killing was done in a similar manner, except the BM Mφs were infected with SPN for 3 hours before rinsing and antibiotic treatment. Percentage of bacteria killed or surviving was calculated utilizing the CFU in the initial inoculum (time zero), which was plated and determined for all experiments. All conditions within each experiment were done in triplicate and all experiments were repeated three times.

### Gram-positive Bacteremia and Gram-negative Endotoxemia Models

Myeloid-specific SIRT1 KO (n = 7) and WT mice (n = 5) were infected with 1 x 10^5^ colony forming units (CFU) of SPN in 400 μL PBS IP as a model of gram-positive bacteremia and sepsis. For a mouse model of gram-negative endotoxemia and shock, mice (n = 5 per group) were injected IP with 20 mg/kg LPS in 200 μl PBS. For all *in vivo* experiments, rectal temperature and survival were assessed every 8 h, and blood was taken retro-orbitally via heparinized capillary tubes at 4 and 24 hours. *In vivo* challenge experiments were repeated twice. 

### Statistics

*In vitro* bactericidal assays were analyzed by one-way ANOVA with Bonferroni post-tests. *In vivo* temperature curves were analyzed by paired one-way ANOVA and Kruskal-Wells post-tests, while ELISA data on serum was analyzed by two-way ANOVA. Mortality was analyzed by one-way ANOVA. Statistical software within GraphPad Prism was used for all analyses.

## Results

### Pharmacologic Alteration of SIRT1 Levels Does not Change Myeloid Cell Killing Capacity

**Figure 1:**
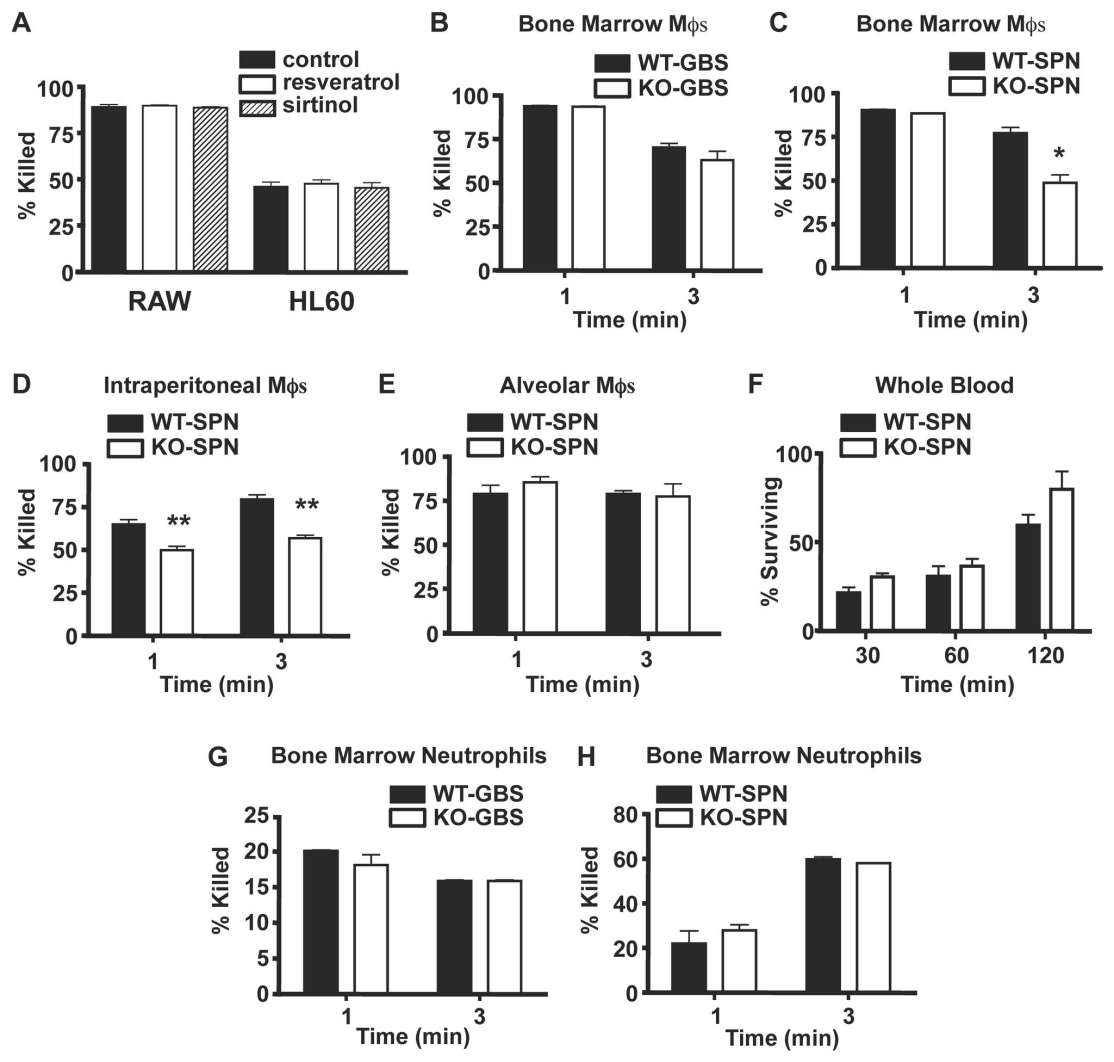
SIRT1 has minimal effects on macrophage and neutrophil antimicrobial function. Addition of a SIRT1 agonist (resveratrol) or antagonist (sirtinol) has no effect on the antimicrobial activity of the mouse derived Mφ cell line RAW 264.7 or the human derived neutrophil-like cell line HL60 (A). Lack of SIRT1 has no effect on BM-derived Mφ killing of GBS (B), but modestly decreases the ability of BM-derived Mφs to kill SPN (C). IP Mφs lacking SIRT1 demonstrate slightly decreased ability to kill SPN (D). SIRT1 deficiency does not affect alveolar Mφ antimicrobial activity (E), or change bacterial survival in whole blood (F). SIRT1 deficiency does not change the antimicrobial activity of BM neutrophils against GBS (G) or SPN (H). KO of SIRT1 in BM-derived Mφs decreases intracellular killing and phagocytosis of SPN (I). Percent bacteria killed or surviving was calculated based on initial inoculum. All conditions were done in triplicate within each assay, and each assay was repeated three times. Data represent mean ± SD, *p < 0.05, **p = 0.003, *** p < 0.001, **** p < 0.0001.

We first sought to define the role of SIRT1 in antimicrobial activities of myeloid cell lines. Murine Mφs (RAW cells) and human neutrophil-like cells (HL60s differentiated with 1.25% DMSO) were infected with GBS, either alone or in the presence of increasing concentrations (5, 10, or 20 μM) of resveratrol, a SIRT1 activator, or sirtinol (10 μM), a SIRT1 inhibitor. At 60 minutes, the RAW and HL60 cells had killed 96% and 47% of the GBS, respectively, and this was not significantly changed by treatment with resveratrol or sirtinol (Figure 1A). Hence, Mφ and neutrophil antimicrobial activity is not markedly affected by pharmacologic activation or inhibition of SIRT1.

### SIRT1 Knock-out Efficiency in SIRT1 LysMcre Mouse Myeloid Cells

qPCR results on total RNA from SIRT1 WT and KO myeloid cells consistently demonstrate a >90% knock-down efficiency in the SIRT1 KO cells. In addition, some SIRT1 KO samples had undetectable levels of SIRT1 RNA, corresponding with a 100% knock-down. Therefore, SIRT1 expression in myeloid cells in C57BL/6 SIRT1 lysMcre mice is between 0-9%. This degree of knock-down is comparable to that found in other lysMcre knock-out mouse strains [1,2].

### SIRT1 KO has Little Effect on Antimicrobial Activities of Macrophages

We next utilized a genetic model to further explore a potential role of SIRT1 in antimicrobial defense. BM, alveolar, and peritoneal Mφs were harvested from WT and SIRT1 myeloid KO mice. These Mφ populations represent various levels of activation, ranging from minimal (BMDM) to highly activated (peritoneal). Alveolar Mφs were used as mature Mφs from a tissue site commonly affected by both GBS and SPN. BMDM from SIRT1 KO mice killed 65% of GBS at 3 hours compared to 72% killed by WT controls (p = ns, Figure 1B). When infected with SPN, SIRT1 KO BMDM showed a significantly reduced level of bacterial killing vs. WT controls (49% vs. 77%, p < 0.05, Figure 1C). Similarly, peritoneal Mφs from SIRT1 KO mice showed a killing defect against SPN bacteria at 1 and 3 hours compared to the WT strains (p < 0.001, Figure 1D). In contrast, no significant defect in SPN killing was observed in alveolar macrophages (Figure 1E). Further, SPN growth was similar in whole blood (Figure 1F). To evaluate the mechanism by which SIRT1 KO BMDM had decreased killing of SPN, we ran intracellular killing and phagocytosis assays. SIRT1 KO BMDM had less intracellular killing of SPN compared to WT (p = 0.0033, Figure 1I), and, as a mechanism, the SIRT1 KO BMDM had decreased phagocytosis compared to WT (p < 0.0001, Figure 1I). We thus conclude loss of SIRT1 has perhaps a modest effect to reduce Mφ antimicrobial activity via decreased phagocytosis, but that this effect depends on the type and origin of Mφ and the bacterial strain. 

### Neutrophil SIRT1 KOs maintain antimicrobial efficacy

The neutrophil is another important cell in host defense, thus we utilized our genetic KO mouse model to study the effect of SIRT1 deficiency on primary neutrophils. BM neutrophils from WT and SIRT1 myeloid KO mice killed GBS and SPN equally well (Figure 1G, 1H). We also observed that antimicrobial activity of peritoneal neutrophils did not differ between WT and SIRT1 myeloid KO mice (data not shown). We thus conclude that SIRT1 has no effect on neutrophil antimicrobial activity, regardless of neutrophil activation state or the bacterial strain, suggesting that therapeutic suppression of SIRT1 may be accomplished without provoking increased bacterial susceptibility.

### Myeloid Cell SIRT1 Deficiency does not Impact Mortality in Models of Gram-negative Endotoxemia or Gram-positive Bacterial Infection

**Figure 2:**
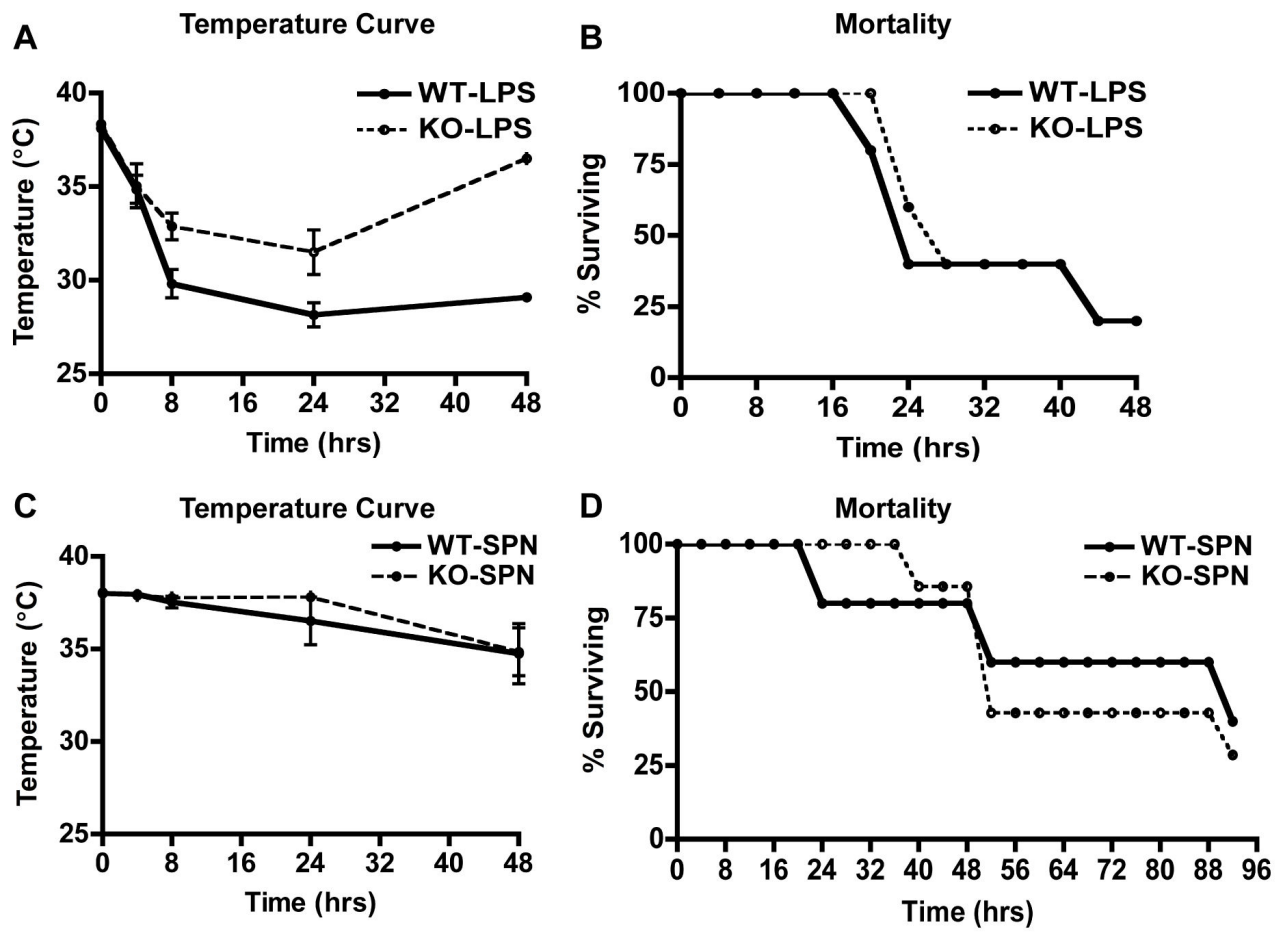
SIRT1 deficiency in myeloid cells has no effect on mortality due to gram-negative endotoxemia or gram-positive bacteremia. SIRT1 myeloid KO mice were more resistant to hypothermia in the face of LPS challenge than WT mice (A), however mortality was identical in the two groups (B; n = 5 mice per group). SIRT1 myeloid KO mice demonstrated no difference in temperature response (C) or mortality (D) due to gram-positive bacterial infection (n = 5 WT and 7 KO mice). *In* *vivo* experiments were repeated twice. Data represent mean ± SD, *p < 0.05.

We next sought to understand the biologic relevance of SIRT1 in innate immune cells during systemic bacterial infections. SIRT1 myeloid KO mice injected with LPS were more resistant to hypothermia than WT mice (31.5°C vs 28.1°C at 24 hours, p < 0.05, Figure 2A). However, this was not associated with any survival benefit, as mortality was identical in the two groups (Figure 2B). Systemic SPN challenge led to similar mortality curves and hypothermia parameters in WT and SIRT1 myeloid KO mice (Figure 2C and 2D). Serum levels of MIP-2, TNFα, and IL-6 were similar at 4 and 24 hours in WT and SIRT1 KO mice treated with LPS (Figure S1A) and infected with SPN (Figure S1B). We conclude that while SIRT1 may play minor role in the bactericidal capacity of certain Mφ populations, overall deletion of SIRT1 in the myeloid lineage has no effect on survival during gram-positive bacteria systemic infection or gram-negative endotoxemia. 

## Discussion

We demonstrate here that pharmacologic activation or inhibition of SIRT1 has no effect on the bacterial killing capacity of mouse- or human-derived Mφ or neutrophil cell lines. We also show that SIRT1 deficiency in IP and BM-derived Mφs modestly decreases their ability to kill SPN, primarily via reduced phagocytosis. SIRT1 expression has no effect on neutrophil antimicrobial activity. Finally, we demonstrate that mice lacking SIRT1 expression in myeloid cells are resistant to endotoxin-induced hypothermia, but succumb to gram-positive bacteremia/sepsis and gram-negative endotoxemia/shock with similar kinetics as WT mice. 

Previous groups have shown that resveratrol decreases LPS-induced TNFα secretion from RAW cells, while sirtinol amplifies the pro-inflammatory response to LPS [28,29]. In contrast, resveratrol has been shown to induce apoptosis of HL60 cells in a dose-dependent manner [30–32]. To our knowledge, ours is the first study to examine the functional effects of either molecule on the bacterial killing capacity of RAW and HL60 cells, and our data indicates that the antimicrobial properties of these cell lines are largely independent of SIRT1 expression.

Little is known about the role of SIRT1 in Mφs, and previous studies have focused on the effects on inflammation rather than on antimicrobial functions. For example, suppression of SIRT1 has been shown to increase Mφ infiltration into inflamed tissues in mouse models of diabetes and colitis [33,34], perhaps by increased NFkB activity [19] and increased expression of MMP9 [35]. Less is know about SIRT1 activity in neutrophils. SIRT1 expression is decreased in neutrophils differentiated from acute promyelocytic leukemia cells [36], suggesting that it may be expressed at low levels in this cell type. 

We utilized the Cre-*loxP* system [37] to target SIRT1 in myeloid cells alone as the global KO of SIRT1 is embryonic lethal [14,38]. Specifically, we utilized SIRT1^flox/flox^ Cre+ mice as our KO and SIRT1^flox/flox^ Cre- mice as our WT control. Theoretically, the loxP sites are within introns and therefore do not interfere with the normal function of the SIRT1 gene. However double-floxing of alleles can have an effect on expression, and thus Cre- mice in a double-floxed mouse may have different findings than a non-floxed C57BL/6 mouse. In addition, Cre expression is driven by the lysozyme promoter and thus should be specific to myeloid cells, thus Cre+ SIRT1^flox/flox^ mice should only be different from Cre- SIRT1^flox/flox^ mice in that they don’t express SIRT1. However, Cre expression alone can affect cell physiology when expressed highly, and possibly could recognize genomic sequences that resemble loxP sites to cause DNA damage. Thus Cre+ non-floxed mice may behave differently than Cre- non-floxed or Cre- SIRT1^flox/flox^ mice (Jackson Labs).

Despite mildly decreasing Mφ antimicrobial activity and affording resistance to endotoxin-induced hypothermia, SIRT1 expression had no effect on mortality due to gram-positive infection or endotoxemia. Redundancy within the sirtuin family may explain this finding. For example, SIRT6 is upregulated in BMDM from SIRT1 KO mice, and is bound to NF-κB transcriptional regulatory elements [17]. Our findings emphasize the importance of examining both *in vitro* and *in vivo* effects of, and both inflammatory and antimicrobial responses to, potential immune mediators such as SIRT1.

SIRT1 is under investigation as a therapeutic target in metabolic, cardiovascular, neurodegenerative and metastatic diseases. Given the emerging medicinal potential of this molecule, we studied its role in host innate defense and have demonstrated that therapeutic suppression of SIRT1 might be accomplished with limited effects on the host myeloid cell response to bacterial infections.

## Supporting Information

Figure S1 **Sirtuin1 lysMcre Cre+ KO (SIRT1 KO) and lysMcre Cre- (WT) control mice were injected with LPS i.p. in a gram-negative model of sepsis.** Mice were bled via accessing the retro-orbital vein at 4 and 24 hours with heparinized capillary tubes. The blood was spun at 1600rpm for 15 minutes and serum was frozen at -80°C until being diluted 1:10 for ELISAs (R&D Systems Duosets). Serum levels of MIP-2, TNFα, and IL-6 were similar at 4 and 24 hours in WT and SIRT1 KO mice (A). Mice were infected with SPN i.p. in a gram-positive model of sepsis. Serum levels of MIP-2, TNFα, and IL-6 were similar at 4 and 24 hours in WT and SIRT1 KO mice (B). All serum samples from individual mice were tested in triplicate.
(TIFF)
