# Supplementary figures and images for: Myeloid Cell Sirtuin-1 Expression Does Not Alter Host Immune Responses to Gram-Negative Endotoxemia or Gram-Positive Bacterial Infection
Source: PLoS One. 2013 Dec 26;8(12):e84481. doi: 10.1371/journal.pone.0084481 (PMC3873454; doi:10.1371/journal.pone.0084481)

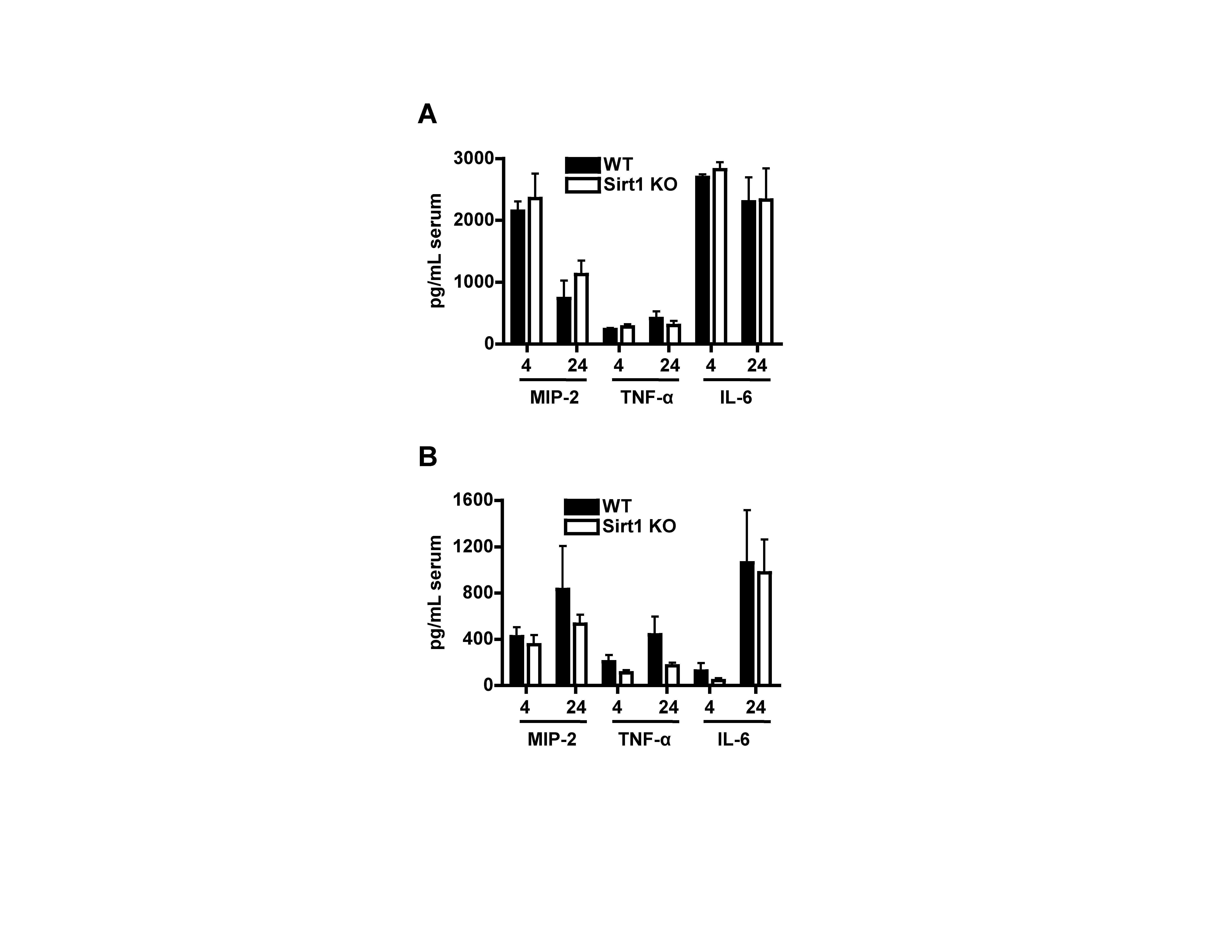

Supplement: Figure S1 — Sirtuin1 lysMcre Cre+ KO (SIRT1 KO) and lysMcre Cre- (WT) control mice were injected with LPS i.p. in a gram-negative model of sepsis. Mice were bled via accessing the retro-orbital vein at 4 and 24 hours with heparinized capillary tubes. The blood was spun at 1600rpm for 15 minutes and serum was frozen at -80°C until being diluted 1:10 for ELISAs (R&D Systems Duosets). Serum levels of MIP-2, TNFα, and IL-6 were similar at 4 and 24 hours in WT and SIRT1 KO mice (A). Mice were infected with SPN i.p. in a gram-positive model of sepsis. Serum levels of MIP-2, TNFα, and IL-6 were similar at 4 and 24 hours in WT and SIRT1 KO mice (B). All serum samples from individual mice were tested in triplicate. (TIFF) [file pone.0084481.s001.tiff]
